# LASSO-based NTCP model for radiation-induced temporal lobe injury developing after intensity-modulated radiotherapy of nasopharyngeal carcinoma

**DOI:** 10.1038/srep26378

**Published:** 2016-05-23

**Authors:** Cheng Kong, Xiang-zhi Zhu, Tsair-Fwu Lee, Ping-bo Feng, Jian-hua Xu, Pu-dong Qian, Lan-fang Zhang, Xia He, Sheng-fu Huang, Yi-qin Zhang

**Affiliations:** 1 Department of Radiation Oncology, Affiliated Hospital of Nanjing Medical University, and Cancer Center of Jiangsu Province, Nanjing, People’s Republic of China; 2 Medical Physics and Informatics Laboratory of Electronics Engineering, National Kaohsiung University of Applied Sciences, Kaohsiung, 80778 Taiwan, ROC; 3 Institute of Clinical Medicine, Kaohsiung Medical University, 807 Taiwan, ROC; 4 Department of Radiology, Affiliated Hospital of Nanjing Medical University, and Cancer Center of Jiangsu Province, Nanjing, People’s Republic of China

## Abstract

We investigated the incidence of temporal lobe injury (TLI) in 132 nasopharyngeal carcinoma (NPC) patients who had undergone intensity-modulated radiotherapy (IMRT) in our hospital between March 2005 and November 2009; and identified significant dosimetric predictors of TLI development. Contrast-enhanced lesions or cysts in the temporal lobes, as detected by magnetic resonance imaging (MRI), were regarded as radiation-induced TLIs. We used the least absolute shrinkage and selection operator (LASSO) method to select D_max_ (the maximum point dose) and the D_1cc_ (the top dose delivered to a 1-mL volume) from 15 dose-volume-histogram-associated and four clinically relevant candidate factors; the D_max_ and the D_1cc_ were the most significant predictors of TLI development. We drew dose-response curves for D_max_ and D_1cc_. The tolerance dose (TD) for the 5% and 50% probabilities of TLI development were 69.0 ± 1.6 and 82.1 ± 2.4 Gy for D_max_ and 62.8 ± 2.2 and 80.9 ± 3.4 Gy for D_1cc_, respectively. The incidence of TLI in NPC patients after IMRT was higher than expected because the therapeutic window is narrow. High-quality longitudinal studies are needed to gain further insight into the complex spatiotemporal effects of non-uniform irradiation on TLI development in NPC patients.

Temporal lobe necrosis is one of the most serious late complications developing after definitive radiotherapy of nasopharyngeal carcinoma (NPC) patients. Such injury has a tremendous impact on both quality-of-life (QoL) and survival in the era of conventional two-dimensional radiotherapy (2D-RT)^1^,^2^,^3^. Currently, the use of intensity-modulated radiotherapy (IMRT) (in preference to 2D-RT or forward-planned 3D-RT) to treat NPC has greatly reduced the occurrence of late toxicities, as IMRT improves sparing of adjacent normal tissues. However, in patients with locally advanced NPC, tumor infiltration into the skull base and intracranial tissue is common. The surrounding normal organs (anteriorly: the optic nerve and chiasm; posteriorly: the brain stem) have relatively low tolerances to radiation, and strict dose constraints must be put in place to preserve their critical physiological functions. High doses to the temporal lobes (TLs), a common undesirable side-effect of treatment, may trigger RT-induced TL injury (TLI). Calculation of appropriate dose thresholds improves treatment planning, thereby mitigating TLI development and preventing the negative impacts thereof on the QoL of locally advanced NPC patients^4^,^5^,^6^.

In recent years, the clinical characteristics of, risk factors for, and dose-volume tolerances appropriate to prevent, TLI after IMRT have been preliminarily explored in several retrospective studies^4^,^5^,^7^,^8^,^9^,^10^. However, significant methodological heterogeneities are evident among these studies, rendering it impossible to compare the results, and the actual tolerance dose (TD) used to avoid the development of TLI after IMRT remains ambiguous. The development of TLI probably depends heavily on dose-volume-histogram (DVH)-associated risk factors. Selection of the most predictive DVH-related factor for incorporation in a risk-estimating model of TDs is technically difficult, because of the general multicollinearity problem of multivariable analysis. Recently, a sophisticated tool, the least absolute shrinkage and selection operator (LASSO), has been used to address the multicollinearity problem effectively and thus to identify important predictors of injury for logistic regression ‘normal-tissue complication probability’ (NTCP) modelling of the lung, parotid gland, small bowel, and oesophagus^11^,^12^, but not the TL yet. Therefore, in the present study, we sought to upgrade the methodology to allow making valid predictions of the tolerance threshold of the TL. We used actuarial-rate-based NTCP modelling and a ‘custom’ LASSO tool to identify critical predictors of TLI development. The incidence and clinical characteristics of TLI in NPC patients treated via IMRT were also investigated. Finally, the methodological quality of the relevant literature was assessed, and we offer recommendations for future studies.

## Materials and Methods

### Patient characteristics

This retrospective study was approved by the ethics committee of the Nanjing Medical University Cancer Center. All participants provided written informed consent, and all experiments were performed in accordance with all relevant guidelines and regulations.

From March 2005 to November 2009, 179 newly diagnosed, biopsy-proven, consecutive NPC patients were treated in our hospital. Of these, MRI data allowing TLI evaluation after completion of IMRT were available for 132. MRI of the other 47 cases had been performed only before the earliest time of TLI occurrence (Supplementary Information, part 1). Thus, we used data from these 132 cases in the analysis. The male/female ratio was 2.7:1, and patient age ranged from 12 to 77 years (median, 47 years). Using the 2010 7^th^ AJCC staging system^13^, 12 patients had stage I disease, 23 stage II, 36 stage III, and 61 stage IVa-b. By T-stage classification, 41 patients were T1, 11 T2, 33 T3, and 47 T4. All patients underwent a series of pre-treatment evaluations and examinations (including history-taking, physical examination, haematological and biochemical profiling, nasopharynx and neck MRI, thoracic-abdominal computed tomography [CT], and whole-body single-photon emission CT bone scanning) to exclude those with contra-indications to treatment and distant metastases.

### Treatment Methods

#### IMRT

Inverse IMRT treatment planning was performed on a Varian Inspiration Platform (version 10.0) using the simultaneous integrated boost technique. The nasopharyngeal primary lesions and their direct extensions (GTVnx) and positive neck lymph nodes (GTVnd) were delineated according to the recommendations of the International Commission on Radiation Units and Measurements Reports nos. 50 and 62. The clinical target volume 1 (CTV1) was defined as the GTVnx with 5–10-mm margins to encompass areas at high risk of microscopic extension and the entire nasopharyngeal mucosa plus a 5-mm depth of sub-mucosal tissue. The CTV2 was defined by addition of 3–10-mm margins to the CTV1 to include areas at low risk of microscopic extension, the level of the identified positive lymph node, and the elective cervical region. The corresponding planning target volumes (PTVs) were generated from the GTVs or CTVs plus 3-mm margins to allow for setup uncertainties. The prescribed doses were 68–75 Gy to the PTV of the GTVnx, in 32–34 fractions; 64–75 Gy to the PTV of the GTVnd, in 32–34 fractions; 60 Gy to the PTV of CTV1, in 32 fractions; and 50 Gy to the PTV of CTV2, in 28 fractions. All patients were given one fraction daily 5 days a week. The DVHs of the organs at risk were evaluated as described in the RTOG0225 protocol, to prevent violation of the tolerance limits^14^.

#### Chemotherapy

IMRT alone was recommended for stage I patients and IMRT combined with concurrent cisplatin/nedaplatin-based chemotherapy for stage II-IV_b_ patients^15^. Neoadjuvant chemotherapy was prescribed for patients with bulky lesions (at the primary site or in the neck); those with residual disease after IMRT received adjuvant chemotherapy.

### Image assessment and the diagnostic criteria for TLI

The endpoint of analysis was the development of TLI as identified by MRI after irradiation. Only contrast-enhanced lesions evident on post-contrast T1-weighted images and cysts were regarded as radiation-induced temporal injuries^16^. All MR images were reviewed independently by two examiners (L.F.Z. and S.F.H.) who specialized in head-and-neck cancer. Consensus was reached by discussion if any initial disagreement was apparent. As both QoL and brain function were of concern, close attention was paid to clinical features relevant to TLI after imaging diagnosis.

### Temporal lobe re-contouring and DVH data collection

As the temporal lobes had been delineated arbitrarily during original IMRT planning, we used a recommended method^17^ to re-contour the temporal lobes. This allowed us to collect accurate data on the following dosimetric parameters: the volume of the TL (TLV), the mean dose, the median dose, the D_max_ (the maximum point dose), the V_40_ (the volume receiving at least 40 Gy; the following five parameters are similar), V_50_, V_60_, V_65_, V_70_, and V_75_, and the D_0.1cc_ (the maximum dose delivered to a volume of 0.1 mL; the following four parameters are similar), D_1cc_, D_5cc_, D_10cc_, and D_20cc_. In addition, the clinical variables age, sex, T stage, and chemotherapy use were included in the analysis.

### Follow-up evaluation

Follow-up included clinical assessment and MRI of the head and neck. The follow-up duration was calculated from the end of IMRT to the day of the final scan. All patients were routinely followed-up at least every 3 months during the first year, every 3–6 months during the next 2 years, and annually thereafter. The usage of MRI examination on the head and neck region during follow up was well implemented. A total of 828 MR images were collected during follow-up; an average of approximately six scans was available for each patient. The TLI latency period was measured from the time of IMRT completion to the first appearance of TLI.

### Statistical Analysis

#### Regularization and variable selection using the elastic net (LASSO)

Two dose-response models (Cox’s regression model and a logistic model) were created to calculate the TLI TDs. The explanatory variables were selected from 15 DVH-based and 4 clinical variables. Strong correlations were evident among candidate variables, as revealed non-parametrically by calculation of Spearman’s rank coefficients, and LASSO was thus performed in a setting of 10-fold cross-validation. This regularization technique identified important predictors of TLI development. Empirical studies have suggested that the elastic net technique can outperform LASSO when the data contain highly correlated predictors. Thus, we set the alpha name-value pair to 0.5 to force LASSO to engage in elastic net regularization. The lambda value of the minimal mean squared error plus one standard deviation was the enforced setting. A free online Matlab package was used to fit the entire elastic-net path for Cox’s regression model^18^.

#### Dose response model

The dosimetric variable most predictive of TLI development, determined using the elastic net, was analysed in the context of a dose response model. Both Cox’s regression and logistic models, using only the most predictive dosimetric variable as an independent variable, were adopted to construct dose-response relationships. In Cox’s dose-effect model, curves adjusted in terms of various values of the dosimetric covariate were plotted to obtain the exact values (TDs) that rendered the failure probabilities at 60 months to be 0.05, 0.1, 0.2, and 0.5, respectively. The logistic dose-effect model was also used to calculate the TDs and to validate the results yielded by Cox’s regression model. The standard errors of the TDs at the different response probabilities were generated using maximum-likelihood estimation.

In the elastic net-fitting and dose-response models, it was assumed that each temporal lobe of a patient was independently affected. Patients who suffered local relapses and underwent repeat RT that delivered an additional dose to a temporal lobe were censored at the time of the repeat RT; this allowed us to evaluate toxicities exclusively attributed to the primary RT. Death from any cause was also regarded as a censoring event if it occurred prior to TLI diagnosis.

Statistical analysis was performed using Stata SE software, version 13.1 (College Station, TX, USA). Group patient characteristics were compared using the χ^2^-test and the t-test. All statistical tests were two-sided, and a P value < 0.05 was considered to reflect statistical significance.

More methodological details can be found in the Supplementary Information.

## Results

### Survival and patterns of treatment failure

The median follow-up duration was 63.5 months (range, 11–106 months). The 5-year overall survival, disease-free survival, and disease-specific survival rates were 84.7%, 74.0%, and 86.9%, respectively. Fifteen (11.4%) patients developed recurrence and 24 (18.2%) developed distant metastases. The median time to recurrence was 25 (range, 11–72) months and that to development of distant metastasis 15.5 (range, 3–90) months. Twenty-three (17.4%) patients died during the follow-up period.

### Incidence and characteristics of TLI

**Figure 1:**
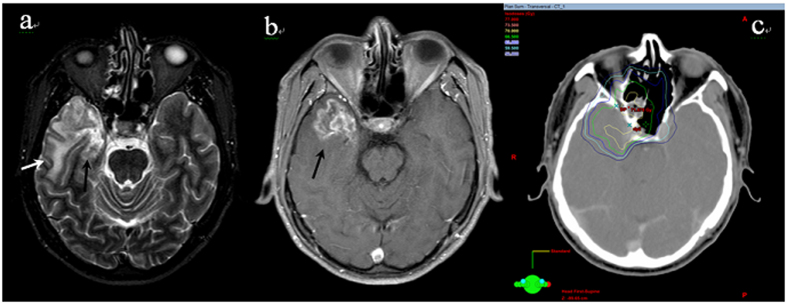
An example of TLI lesion showing (**a**) homogeneous high signal area in the white matter on T2-weighted images (white arrow) and the medial necrosis (black arrow) (**b**) contrast-enhanced lesion on postcontrast T1-weighted MR images with internal necrosis (black arrow) (**c**) corresponding isodose lines.

**Figure 2:**
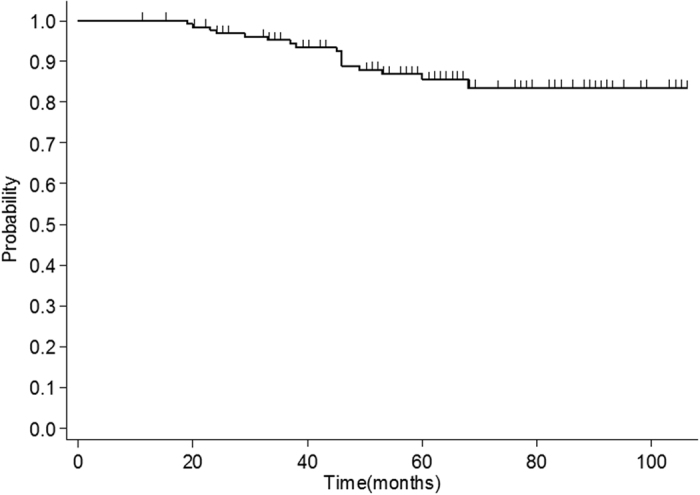
The probability of the TLI-free survival (n = 132) is shown. Each censored case is indicated by a spike.

A total of 17 cases (17/132, 12.9%, crude rate) developed MRI-indicated TLI. The baseline characteristics of these patients are summarized and compared with those of patients without TLI in Table 1. Of the 17 cases, 13 (76.5%) and 4 (23.5%) had radiation-induced injuries to the unilateral and bilateral TLs, respectively. Two cases simultaneously developed radiation-induced brain stem damage (contrast-enhanced lesions were evident on post-contrast T1-weighted images). Of the 17 patients who developed TLIs, 8 exhibited varying degrees of clinical symptoms, such as vertigo, headache, memory deterioration, muscle weakness, and personality changes; the remaining 9 patients were asymptomatic. Overall, the MRI findings in the TLI patients revealed a continuous spectrum of RT-associated damage. Small solid enhanced nodules were evident in five cases, while moderate and large lesions were apparent in seven and five cases, respectively (lesions were classified as described in ref. 16). The outcomes of TLI evolution were documented; the MRI features of TLI (including signal abnormality on T1 and T2 images) resolved completely in four patients following gradual improvement over a few years. In contrast, one patient developed a large contrast-enhanced lesion, with a central necrotic core, in the right TL, accompanied by severe white matter lesions with a large confluent area that extended superiorly and caused a mass effect. This was the only patient to die from TLI. Apart from one T2 case who had not received chemotherapy, all other TLI patients were of stage T3 (6 cases) or T4 (10 cases) and underwent chemotherapy during their treatment periods. Data from a representative TLI case, and the corresponding dose distributions, are shown in Fig. 1. The median TLI latency period was 43 months (range, 19–68 months). The overall TLI-free survival rate was calculated using the Kaplan-Meier procedure (Fig. 2); the actuarial TLI-free survival rates were 95.5% and 85.8% at 3 and 5 years, respectively.

### Selection of predictors and estimation of tolerance doses

The maximum, minimum, and mean values of 15 DVH-associated variables are listed in Table 2 by subgroup (with or without TLI). Elastic net fitting of these 19 variables revealed that only 4 (D_max_, D_0.1cc_, D_1cc_, and T stage) were selected (Figs s1 and s2). Based on prior knowledge^4^,^5^,^10^,^19^ and the law of parsimony, we preferred to use D_max_ and D_1cc_ to estimate TLI risks employing single DVH point thresholds in univariate logistic/Cox’s dose-response models.

#### Cox’s model

**Figure 3:**
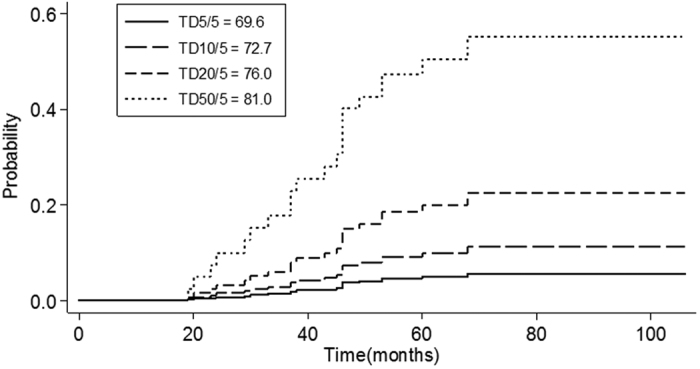
Failure functions adjusted to D_max_ using a univariate Cox model (n = 264) are shown. The failure functions adjusted to D_1cc_ were similar and omitted.

A global test using the Schoenfeld residuals indicated that the PH assumption was not violated. Univariate Cox’s regression showed that both D_max_ and D_1cc_ were significant risk factors (P < 0.001). Thus, we evaluated D_max_ and D_1cc_ individually in Cox’s dose-response model. The 5-year TLI incidences were predicted to be 5, 10, 20, and 50% for D_max_ values of 69.6, 72.7, 76.0, and 81.0 Gy, respectively. For those D_1cc_ values, the doses were 63.4, 67.9, 72.5, and 79.6 Gy, respectively (Fig. 3).

#### Logistic modelling

Sun *et al.*^8^ found that 19/20 (95%) TLI cases had latencies of less than 50 months, and in the present study, 16/17 (94%) of the TLI cases had latencies of less than 50 months. Thus, injury-free TLs evident on MRI for more than 50 months were regarded as normal. Cases censored because of death or repeat RT within 50 months were excluded because of the need for adequate follow-up to determine whether the TLs would develop radiation injuries.

**Figure 4:**
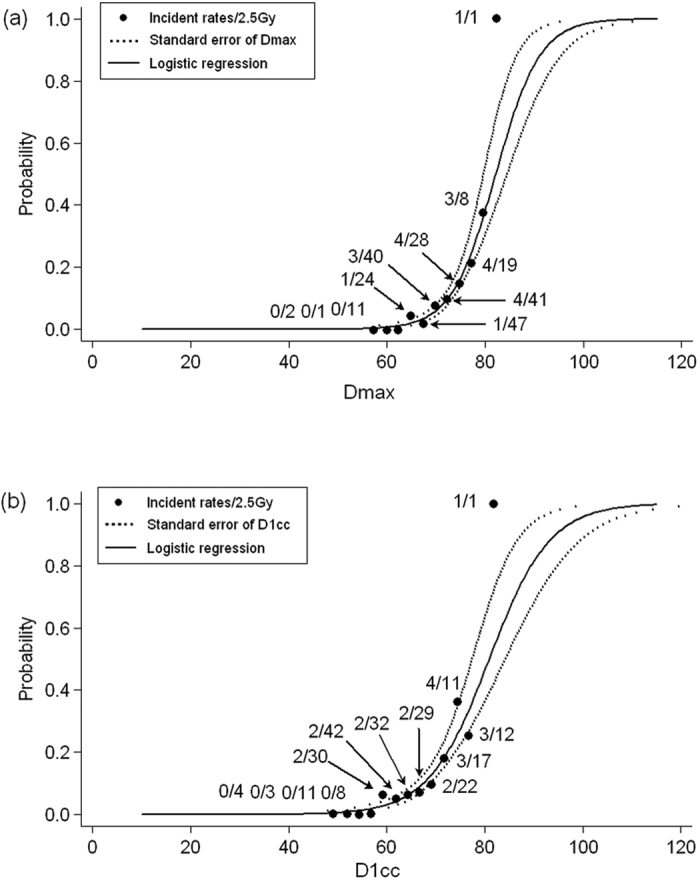
Dose effect curves using a logistic model (n = 222) (**a**) for variable D_max_ and (**b**) variable D_1cc_. Dotted lines indicate the single standard error of the tolerance dose at discrete probabilities of 0.01 to 0.99.

Consequently, the 21 cases censored within 50 months because of death or repeat RT were excluded, 42 TLs were excluded, and 222 TLs were subjected to analysis. Using the two input variables selected from the elastic net, the results of univariate logistic regression were consistent with the findings of Cox’s regression, namely, that both D_max_ and D_1cc_ were significant predictors of injury. Thus, either D_max_ or D_1cc_ was adopted as the best parsimonious predictor in logistic dose-response modelling. The TDs at different response probabilities are shown in Table 3 and are consistent with the dose constraints acquired from Cox’s model. To allow visualization, the fitted dose-response curves and the superior and inferior standard error bounds are plotted together with the crude incidence rates calculated at discretized dose intervals of 2.5 Gy (Fig. 4).

## Discussion

Although IMRT has been widely used to treat NPC over the past decade, neither the incidence of TLI after IMRT nor the TD have been well-defined, and accurate data are required for clinical planning. Recently, in two early studies of IMRT-induced TLI in NPC patients treated at a single centre, the authors discussed their experiences with dosimetric features associated with the occurrence of TLI^4^,^7^. However, non-ignorable selection biases were evident in both studies; the inclusion criteria for injured and normal TLs were not balanced (normal TL, no injury on follow-up for ≥60 months; injured TL, injury present for as long as injury was noted). In essence, the use of crude rates can underestimate the risks, causing deviations in TD calculations, attributable to inaccurate TLI rates^20^. Therefore, we recommend the use of actuarial-rate-based approaches for TD estimation. This indicates that Cox’s model must be adjusted in terms of the specified independent variable used to calculate tolerance. Such methods may potentially be useful additions to typical NTCP modelling. Our work may represent an initial step in the development of a statistically rigorous method for estimation of TDs.

Radiation dose-response curves are typically sigmoid in shape, and NTCP models such as the logistic and Lyman models are frequently used to construct such curves^21^. Empirically, the sigmoid-shaped NTCP model applied in our present study and in ref. 10 is superior to the linear model used in refs 4 and 7 for data-fitting and may therefore provide more precise estimations. It is also important to consider multicollinearity effects, which were not addressed in most studies featuring multivariable analyses of dosimetric risk factors for TLI^4^,^5^,^7^,^8^,^9^. As most dosimetric predictors are highly correlated, direct insertion of DVH-based parameters into a multivariate model may not achieve meaningful results. The most recent study used principal component analysis (PCA) to select variables (in a pre-processing step) and employed a logistic NTCP model similar to ours to calculate TDs^10^. However, as we saw in ref. 10, the extensive multicollinearity evident among dosimetric parameters was avoided by manually selecting D_1cc_/V_D>80_ to represent all other correlated factors, rather than the efficiency of PCA per se. However, it is questionable whether the D_1cc_/V_D>80_ is representative of all information in the cluster. Thus, as PCA is intrinsically defective, we applied elastic net fitting to Cox’s regression model to identify the crucial factors. The methodological limitations of recent reports on TL tolerance doses in NPC patients treated via IMRT are listed in Table 4. Considerable methodological heterogeneities are evident among the studies, and therefore, it is very difficult to compare the results. Harmonization of the methodology using actuarial-rate-based NTCP modelling and employing effective predictor-selection tools may be warranted in future studies of TD calculation.

In our current series, the crude TLI rate after IMRT was 12.9% (17/132), higher than the values in recent reports (~7.5%)^5^,^6^. The principal reason is that the entire consecutive cohort served as the denominator; this included some patients for whom MRI data were inadequate for use. This also partially explains the relatively higher biologically effective dose to the TL (e.g., D_max_: 70.5 Gy in 33 fractions, on average) noted in the present study. On the other hand, the rate increase may be partially attributed to the fact that the MRI data is relative sufficient during follow-up in our present study. The TLI of the four cases in whom MRI abnormalities disappeared over a few years may have been overlooked if the intervals between MRI scans had been excessively long. In addition, heterogeneities in terms of patient characteristics, event definitions, prescribed total doses and fractionation doses for tumor treatment^22^, target delineation strategies, TL constraints in the original plans, and chemotherapy all affect the crude and actuarial incidences to some extent. A focus on the dosimetric parameters of the TL per se avoids the need to consider some of these factors, such as the prescribed total and fractionation doses. The dosimetric parameters of the TL per se are better predictors of TLI than are tumor parameters.

Furthermore, we have shown that the NPC therapeutic window is extremely narrow. The dose constraints for D_max_ in Table 3 clearly indicate that the risk of TLI development increases from 1 to 5% with a TD increment of 7.3 Gy and from 5 to 10% with a dose increment of only 3.4 Gy. Thus, an additional boost of 5 Gy in two fractions towards the end of a basic RT course of 70 Gy with 2-Gy daily fractions may dramatically exacerbate the TLI risk^23^. The “narrow therapeutic window” is well-explained by the TL NTCP curve (Fig. 4a). At approximately 70 Gy, the slope increases significantly within a relatively small interval. As a result, a planner should be prudent to provide any additional boost to the primary tumor after the TL D_max_ has reached approximately 70 Gy. If it is difficult to suppress the maximum dose to less than 70 Gy in patients with locally advanced disease, then the TL D_1cc_ or the volume receiving the high dose^9^ should be maximally reduced to minimize the extent of TLI, if it would occur.

Our study had several limitations, including its retrospective study design, heterogeneities in chemotherapy regimens, and a relatively small sample size and event number; these may have limited the statistical power. Another problem was the ad-hoc nature of follow-up MRI, which was performed primarily to detect possible locoregional recurrences rather than TLI. Notably, 9/17 patients with TLIs evident on MRI were asymptomatic. Unless all included patients undergo (at least) annual MRI, the TLI rate will remain under-reported, and dosimetric calculations may thus be in error. Such deficiencies are evident in all relevant retrospective articles, although we made an effort to increase the MRI follow-up frequency in the present study. Ideally, a prospective multicentre study should confirm our data before any definite conclusions are drawn. Given the long latency and relative rarity of overt TLI, the best case scenario, at present, would be retrospective analysis of data from large cohorts of NPC patients treated both prospectively and uniformly in clinical trials. However, our report is useful, because it identifies a valuable direction for future research.

## Additional Information

**How to cite this article**: Kong, C. *et al.* LASSO-based NTCP model for radiation-induced temporal lobe injury developing after intensity-modulated radiotherapy of nasopharyngeal carcinoma. *Sci. Rep.* **6**, 26378; doi: 10.1038/srep26378 (2016).

## Supplementary Material

Supplementary Information

## Figures and Tables

**Table 1:** The base-line characteristics of the patients with and without temporal lobe injury (TLI).

Characteristic	Group without TLI n = 115 (%)	Group with TLI n = 17 (%)	*P*-value
Age (years)			0.15
Median	46	50	
Range	12–77	35–65	
Gender			1.0
Men	83 (72.2)	13 (76.5)	
Women	32 (27.8)	4 (23.5)	
Stage			0.04
I-II	34 (29.6)	1 (5.9)	
III- IVa-b	81 (70.4)	16 (94.1)	
T category			0.002
T1-2	51 (44.4)	1 (5.9)	
T3-4	64 (55.7)	16 (94.1)	
Chemotherapy			1.0
Yes	98 (85.2)	15 (88.2)	
No	17 (14.8)	2 (11.8)	

**Table 2:** Characteristics of the dose-volume variables for the two subgroups (n = 264).

Variable	Variable value for patients without TLI			Variable value for patients with TLI		
	Mean	Minimum	Maximum	Mean	Minimum	Maximum
D_max_	70.1	56.1	80.8	74.3	65.3	83.1
Mean dose	22.7	6.9	47.2	27.3	19.1	37.7
D_0.1cc_	68.3	54.5	79.1	72.7	63.9	82.5
D_1cc_	64.5	48.1	77.4	69.9	58.8	81.1
D_5cc_	53.3	28.1	75.9	62.0	42.9	77.2
D_10cc_	43.4	14.6	74.8	52.9	32	73.3
D_20cc_	32.6	8.2	71.9	39.8	24.2	55.3
TLV	100.2	67	137.8	99.5	70.3	122.2
V_75_	0.2	0	9.0	1.0	0	8.4
V_70_	1.0	0	23.6	3.0	0	12.2
V_65_	2.3	0	30.6	5.1	0.003	14.9
V_60_	4.0	0	36.5	7.3	0.8	17.5
V_50_	8.0	0.8	48.4	12.2	3.1	23.2
V_40_	13.6	2.1	60.1	19.9	6.0	33.5
Median dose	18.9	3.3	45.9	22.4	13.6	31.3

**Table 3:** Tolerance dosages for the specified effect probability levels*.

Tolerance doses	TD for D_1cc_	TD for D_max_
TD_1_	52.7 ± 4.5	61.7 ± 3.2
TD_5_	62.8 ± 2.2	69.0 ± 1.6
TD_10_	67.4 ± 1.5	72.4 ± 1.1
TD_20_	72.4 ± 1.7	76.0 ± 1.2
TD_30_	75.7 ± 2.3	78.4 ± 1.6
TD_50_	80.9 ± 3.4	82.1 ± 2.4

^*^Note that the tolerance doses were referred to with an underlying background of average of 33 fractions in the model.

**Table 4:** The methodological quality of the published literature regarding the tolerance dose of temporal lobe in NPC patients after IMRT.

	Consecutive cohort	Multicolinearity effect	Actuarial rate-based	Selection bias	NTCP model
Ref. 4	Yes	Direct selection (D_max_/D_1cc_) no MA	No	Yes	Linear
Ref. 7	Yes	Not considered in MA	No	Yes	Linear
Ref. 5	Yes	Not considered in MA	No	Unknown	Not used
Ref. 8	No (only TLI cases)	Not considered in MA	No	—	Not used
Ref. 9	No (1:1 match)	Perform MA for each DVH parameter	No	Yes, serious, maybe	Not used
Ref. 10	Yes	using principal component analysis	No	Yes	Logistic
This study	Yes	using Elastic Net(LASSO)	Yes	No	Logistic/cox

Abbreviations: MA = multivariable analysis; NTCP = normal tissue complication probability; TLI = temporal lobe injury; DVH = dose volume histogram; LASSO = the least absolute shrinkage and selection operator.

## References

[b1] Lee A. W. *et al.* Factors affecting risk of symptomatic temporal lobe necrosis: significance of fractional dose and treatment time. Int J Radiat Oncol Biol Phys 53, 75–85 (2002). 12007944 10.1016/s0360-3016(02)02711-6

[b2] Tang Y. *et al.* Clinical characteristics and changes in living quality of patients with radiation encephalopathy induced by radiation therapy for treating nasopharyngeal carcinoma. Neural Regen Res 2, 99–102 (2007).

[b3] Cheung M. C. *et al.* Impact of radionecrosis on cognitive dysfunction in patients after radiotherapy for nasopharyngeal carcinoma. Cancer 97, 2019–26 (2003). 12673733 10.1002/cncr.11295

[b4] Su S. F. *et al.* Clinical and dosimetric characteristics of temporal lobe injury following intensity modulated radiotherapy of nasopharyngeal carcinoma. Radiother Oncol 104, 312–6 (2012). 22857858 10.1016/j.radonc.2012.06.012

[b5] Zeng L. *et al.* Late toxicities after intensity-modulated radiotherapy for nasopharyngeal carcinoma: patient and treatment-related risk factors. British Journal of Cancer 110, 49–54 (2014). 24253503 10.1038/bjc.2013.720 PMC3887308

[b6] Zhou G. Q. *et al.* Radiation-induced temporal lobe injury for nasopharyngeal carcinoma: a comparison of intensity-modulated radiotherapy and conventional two-dimensional radiotherapy. PLos One 8, e67488 (2013). 23874422 10.1371/journal.pone.0067488 PMC3707870

[b7] Su S. F. *et al.* Analysis of dosimetric factors associated with temporal lobe necrosis (TLN) in patients with nasopharyngeal carcinoma (NPC) after intensity modulated radiotherapy. Radiat Oncol 8, 17 (2013). 23336282 10.1186/1748-717X-8-17 PMC3573909

[b8] Sun Y. *et al.* Radiation-induced temporal lobe injury after intensity modulated radiotherapy in nasopharyngeal carcinoma patients: a dose-volume-outcome analysis. BMC Cancer 13, 397 (2013). 23978128 10.1186/1471-2407-13-397 PMC3851326

[b9] Zhou X. *et al.* Effect of Dosimetric Factors on Occurrence and Volume of Temporal Lobe Necrosis Following Intensity Modulated Radiation Therapy for Nasopharyngeal Carcinoma: A Case-Control Study. Int J Radiation Oncol Biol Phys 90, 261–9 (2014). 10.1016/j.ijrobp.2014.05.036 25066214

[b10] Zeng L. *et al.* Normal tissue complication probability model for radiation-induced temporal lobe injury after intensity-modulated radiation therapy for nasopharyngeal carcinoma. Radiology 141721 (2015). 10.1148/radiol.14141721 25658039

[b11] Lee T.-F. *et al.* LASSO NTCP predictors for the incidence of xerostomia in patients with head and neck squamous cell carcinoma and nasopharyngeal carcinoma. Sci Rep 4, 6217 (2014). 25163814 10.1038/srep06217 PMC5385804

[b12] Lee T.-F. *et al.* Patient- and therapy-related factors associated with the incidence of xerostomia in nasopharyngeal carcinoma patients receiving parotid-sparing helical tomotherapy. Sci Rep 5, 13165 (2015). 26289304 10.1038/srep13165 PMC4542473

[b13] Pharynx (Including Base of Tongue, Soft Palate, and Uvula) In AJCC Cancer Staging manual 7th edn. (eds Edge S. B. Fritz A. G. Byrd D. R. Greene F. L. Compton C. C. Trotti A. ) 41–56 (Springer, 2010).

[b14] Lee N. *et al.* Intensity-modulated radiation therapy with or without chemotherapy for nasopharyngeal carcinoma: radiation therapy oncology group phase II trial 0225. J Clin Oncol 27, 3684–90 (2009). 19564532 10.1200/JCO.2008.19.9109 PMC2720082

[b15] Xu J. *et al.* Concurrent chemoradiotherapy with nedaplatin plus paclitaxel or fluorouracil for locoregionally advanced nasopharyngeal carcinoma: Survival and toxicity. Head Neck 36, 1474–80 (2014). 23996842 10.1002/hed.23487

[b16] Wang Y.-X. *et al.* Evolution of Radiation-induced Brain Injury: MR Imaging-based study. Radiology 254, 210–8 (2010). 20019142 10.1148/radiol.09090428

[b17] Baxi S. *et al.* Temporal changes in IMRT contouring of organs at risk for nasopharyngeal carcinoma – The learning curve blues and a tool that could help. Technology in Cancer Research and Treatment 8, 131–40 (2009). 19334794 10.1177/153303460900800206

[b18] Qian J. Hastie T. Friedman J. Tibshirani R. & Simon N. *Glmnet for Matlab*. (2013) Available at: http://www.stanford.edu/~hastie/glmnet_matlab/. (Accessed: 10th January 2016).

[b19] Schlampp I. *et al.* Temporal lobe reactions after radiotherapy with carbon ions: incidence and estimation of the relative biological effectiveness by the local effect model. Int J Radiat Oncol Biol Phys 80, 815–23 (2011). 20638186 10.1016/j.ijrobp.2010.03.001

[b20] Lawrence Y. R. *et al.* Radiation dose-volume effects in the brain. Int J Radiat Oncol Biol Phys 76, S20–7 (2010). 20171513 10.1016/j.ijrobp.2009.02.091 PMC3554255

[b21] Bentzen S. M. Dose-response relationship in radiotherapy In Basic clinical radiobiology 4^th^ edn. (eds Michael J. & Albert van der K. ) 57 (Arnold, 2009).

[b22] Bakst R. L. *et al.* Hypofractionated dose-painting intensity modulated radiation therapy with chemotherapy for nasopharyngeal carcinoma: a prospective trial. Int J Radiat Oncol Biol Phys 80, 148–53 (2011). 20605352 10.1016/j.ijrobp.2010.01.026 PMC2952060

[b23] Lee A. W. *et al.* Major late toxicities after conformal radiotherapy for nasopharyngeal carcinoma—patient- and treatment-related risk factors. Int J Radiat Oncol Biol Phys 73, 1121–8 (2009). 18723296 10.1016/j.ijrobp.2008.05.023

