# Supplementary material for: LASSO-based NTCP model for radiation-induced temporal lobe injury developing after intensity-modulated radiotherapy of nasopharyngeal carcinoma
Source: Sci Rep. 2016 May 23;6:26378. doi: 10.1038/srep26378 (PMC4876467; doi:10.1038/srep26378)
Supplement: Supplementary Information [file srep26378-s1.pdf]

LASSO-based NTCP model for radiation-induced temporal lobe injury developing after intensity-modulated radiotherapy of nasopharyngeal carcinoma

Cheng Kong, Xiang-zhi Zhu, Tsair-Fwu Lee, Ping-bo Feng, Jian-hua Xu, Pu-dong Qian, Lan-fang Zhang, Xia He, Sheng-fu Huang, Yi-qin Zhang

An introduction to the method

1. Of the 179 patients, 132 had MRI examination results available for TLI evaluation after the completion of IMRT. The rest 47 cases had performed MRI only before the earliest time of TLI occurrence. Thus, we discard the 47 cases with 132 cases subjected to the model for analysis. All of the characteristics and results reported in the following sections are based upon N=132. This is a key point for this study. We will give several reasons for why we do this.

All the 47 cases were censored before the earliest failure time of **19 months** after the completion of IMRT. Just for this reason, removing the 47 cases censored before the earliest failure time has no effect on the results, not only for KM analysis, but also for Cox model and logistic model. Here are several examples which can give a justification of it. The data of this example below derive from a study of the remission times in weeks for a group of leukemia patients after treatment.

Remission times (weeks) for a group of leukemia patients after treatment. (n=23)
6, 6, 6, 7, 10, 13, 16, 22, 23,
2+, 3+, 6+, 9+, 10+, 11+, 17+, 19+, 20+, 25+, 32+, 32+, 34+, 35+. (Note: +denotes censored, and two cases have been censored before the earliest failure time of 6 weeks.)

The table below shows the estimated survival probabilities using the Kaplan-Meier formula for this group of patients.

$t_{(j)}$	n_j	m_j	q_j	$\hat{S}(t_{(j)})$
0	23	0	2	1
6	21	3	1	1×(18/21)=.8571
7	17	1	1	.8571×(16/17)=.8067
10	15	1	2	.8067×(14/15)=.7529
13	12	1	0	.7529×(11/12)=.6902
16	11	1	3	.6902×(10/11)=.6275
22	7	1	0	.6275×(6/7)=.5378
23	6	1	5	.5378×(5/6)=.4482

t(j) denotes ordered failure times, n_j gives the number of subjects in the risk set at the start of the interval [t(j) t(j+1)], m_j gives the number of failures during the interval [t(j) t(j+1)], q_j gives the number of censored cases in [t(j) t(j+1)].

Given that the risk set is defined as the collection of individuals who have survived at least to time t(j), it is assumed that n_j includes those persons failing at time t(j). In other words, n_j counts those subjects at risk for failing instantaneously prior to time t(j).

Then, if we deleted the two cases censored before the earliest failure time of 6 weeks, what will happen?

$t_{(j)}$	n_j	m_j	q_j	$\hat{S}(_{t(j)})$
0	21	0	0	1
6	21	3	1	1×(18/21)=.8571
7	17	1	1	.8571×(16/17)=.8067
10	15	1	2	.8067×(14/15)=.7529
13	12	1	0	.7529×(11/12)=.6902
16	11	1	3	.6902×(10/11)=.6275
22	7	1	0	.6275×(6/7)=.5378
23	6	1	5	.5378×(5/6)=.4482

Except for two underlined red figures in the first row of the table, there is practically no difference for the estimated survival probabilities. So we can derive a conclusion that removing the cases censored before the earliest failure time has no effect on the estimated survival probabilities for KM analysis.

It is also the case in the Cox model setting. To illustrate this connection, consider the dataset shown below. The data indicate that Barry got the event at TIME = 2 years. Gary got the event at 3 years, Harry was censored at 5 years, and Larry got the event at 8 years. Furthermore, Barry and Larry were smokers whereas Gary and Harry were nonsmokers.

ID	TIME	STATUS	SMOKE
Barry	2	1	1
Gary	3	1	0
Harry	5	0	0
Larry	8	1	1

Consider the Cox proportional hazards model with one predictor, SMOKE. Under this model the hazards for Barry, Gary, Harry, and Larry can be expressed as shown below.

Cox PH model

$h(t)=h_0(t)\exp(\beta_1 SMOKE)$	
ID	Hazard
Barry	$h_0(t)\exp(\beta_1)$
Gary	$h_0(t)\exp(0)$
Harry	$h_0(t)\exp(0)$
Larry	$h_0(t)\exp(\beta_1)$

The Cox likelihood for these data is shown below:

Likelihood is product of 3 terms

$L=L1\times L2\times L3$

$L1=h_0(t)\exp(\beta_1)/[h_0(t)\exp(\beta_1)+h_0(t)\exp(0)+h_0(t)\exp(0)+h_0(t)\exp(\beta_1)]$

$L2=h_0(t)\exp(0)/[h_0(t)\exp(0)+h_0(t)\exp(0)+h_0(t)\exp(\beta_1)]$

$L3=h_0(t)\exp(\beta_1)/h_0(t)\exp(\beta_1)$

Cox likelihood

$L=\{h_0(t)\exp(\beta_1)/[h_0(t)\exp(\beta_1)+h_0(t)\exp(0)+h_0(t)\exp(0)+h_0(t)\exp(\beta_1)]\}\times$
$\{h_0(t)\exp(0)/[h_0(t)\exp(0)+h_0(t)\exp(0)+h_0(t)\exp(\beta_1)]\}\times\{h_0(t)\exp(\beta_1)/h_0(t)\exp(\beta_1)\}$

To summarize, the likelihood in our example consists of a product of three terms (L1, L2, and L3) corresponding to the ordered failure times (t_1, t_2, and t_3). The denominator for the term corresponding to time t_j (j=1, 2, 3) is the sum of the hazards for those subjects still at risk at time t_j, and the numerator is the hazard for the subject who got the event at t_j.

When the dataset is added with a case named ‘Marry’ (see below), who censored at 1 year before the earliest failure time of 2 years, what will happen?

ID	TIME	STATUS	SMOKE
Marry	1	0	1
Barry	2	1	1
Gary	3	1	0
Harry	5	0	0
Larry	8	1	1

According to the way of how the Cox likelihood function was constructed aforementioned, the likelihood function for the new data is exactly same with the old one. So we can derive that the deletion of cases censored before the earliest failure time has no effect on the likelihood function for Cox model.

When referring to the logistic model, we have defined that the injury-free TLs that followed-up for more than 50 months were regarded as normal and cases censored within 50 months were excluded because of the need of adequate follow-up to determine whether TLs would develop radiation injury. Thus, the 47 cases censored before the earliest failure time of 19 months were destined to be removed according to the definition. As a result, the deletion of cases censored before the earliest failure time has no effect on the logistic analysis.

2. Reference 11 was mentioned in the manuscript (**Results**, **Logistic model** section**)** to make preparation for the following application of logistic model. The response variable in logistic model is binomial, which means a patient has to be defined alternatively as having TLI or normal. Then a problem is closely followed: a patient without TLI (injury-free, not experience the event) until the end of the study is virtually normal? It is easy to understand that TLI has certain latency and the patient with short follow-up may develop TLI later than the end point of the study. So an adequate follow-up is required to determine whether a patient is normal or not. Then, how long the follow-up is adequate? **We utilized the data of reference 11 in combination with our data to raise an available criteria that following-up for more than 50 months is adequate to regard the injury-free temporal lobe as normal.** In the practical situation, clinical follow-up data often includes censored cases, and some patients may not have adequate follow-up to determine the real outcome. The standard statistical method to address this problem is survival analysis, so we recommend using Cox model as a NTCP model. However, in the case of not using survival analysis, e.g. using logistic model, how to do? For the undefined outcome of the injury-free patients with short follow-up, it seems to be plausible to remove the injury-free cases with short follow-up and retain the injury-free cases with adequate follow-up as well as retain all the TLI cases (no matter the length of follow-up). This is the way to take in reference 4 and 5, but an underlying selection bias occurs. We have discussed this point briefly **in the first paragraph**, **Discussion** section. Here we give some details.

In reference 4 and 5, the author presumed injury-free TLs that were followed-up for more than 60 months were regarded as normal, while 18 out of 33 patients of unilateral TLI had a follow-up of less than 60 months. Thus, the authors concluded that the outcome of 18 contra-lateral uninjured TLs could not be determined, and the 18 uninjured TLs were excluded from the analysis. Indeed, if 18 contra-lateral uninjured TLs need to be excluded for a follow-up of less than 60 months, then the 18 injured TLs should also be excluded. To determine the actual incidence rate without using survival analysis (cox model), the sample of TLI cases and normal cases should be included according to the same length of follow-up (e.g., more than 50 months). The use of inconsistent inclusion criteria to select injured cases (no matter the length of follow-up, as long as it occurs) and normal cases (follow-up$\geqslant$50 mo.) that were pooled to determine incidence can result in a non-ignorable selection bias, thereby affecting the TD calculation. It's important that the inclusion criterion for normal and injured cases is identical, e.g. follow-up more than 50 months. Therefore, we recommend an actuarial-rate-based approach to estimate TD, which means the method of Cox model adjustment for the specified independent variable to calculate tolerance.

3. Here we give more information of the software package and algorithm for LASSO and elastic-net. The original sources locate on the website:
http://web.stanford.edu/~hastie/glmnet_matlab/index.html

Glmnet is an algorithm package on Matlab platform which provides the extremely efficient procedures for fitting a generalized linear model via penalized maximum likelihood. The regularization path is computed for the lasso or elasticnet penalty at a grid of values for the regularization parameter lambda. Glmnet can deal with all shapes of data, including very large sparse data matrices. The generalized linear models fitted include linear, logistic and multinomial,

Poisson, and **Cox regression models**. In this study, we use Glmnet to fit **Cox regression model.**

Here is a brief introduction of the package.
Suppose X is the input matrix and Y the response vector. For the families except Gaussian, glmnet maximizes the appropriate penalized log-likelihood (**partial likelihood for the cox model**), or minimize the penalized negative one. Take the binomial model for example, it solves

$$\min_{(\beta_0,\beta)\in\mathbb{R}^{p+1}} -\frac{1}{N}\sum_{i=1}^{N} y_i\cdot(\beta_0+x_i^T\beta)+\log(1+e^{(\beta_0+x_i^T\beta)})+\lambda\left[(1-\alpha)||\beta||_2^2/2+\alpha||\beta||_1\right].$$

Where lambda⩾0 is a complexity parameter and 0⩽alpha⩽1 is a compromise between ridge and lasso. Note that it becomes the lasso when alpha = 1 and the ridge regression when alpha = 0. We set alpha=0.5 to make LASSO provide elastic net regularization. The algorithm uses cyclical coordinate descent in a pathwise fashion.

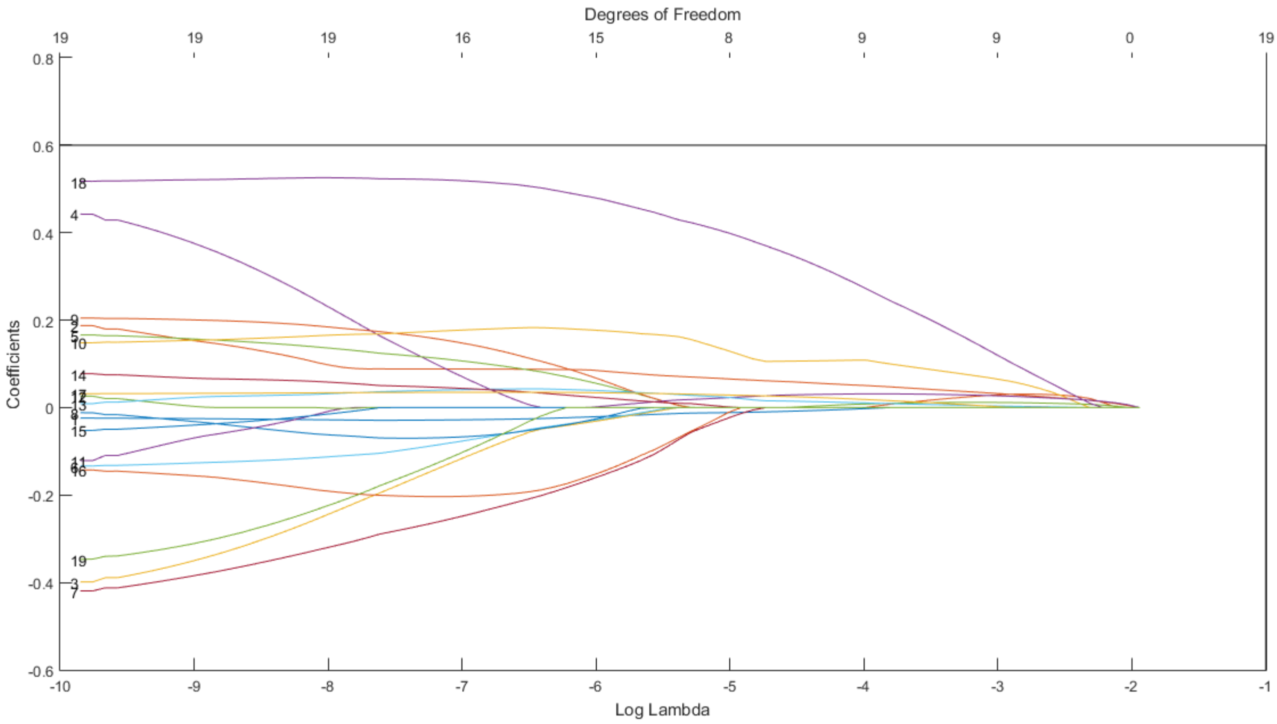

Fig. s1. Trace plot of coefficients fit by the elastic net.

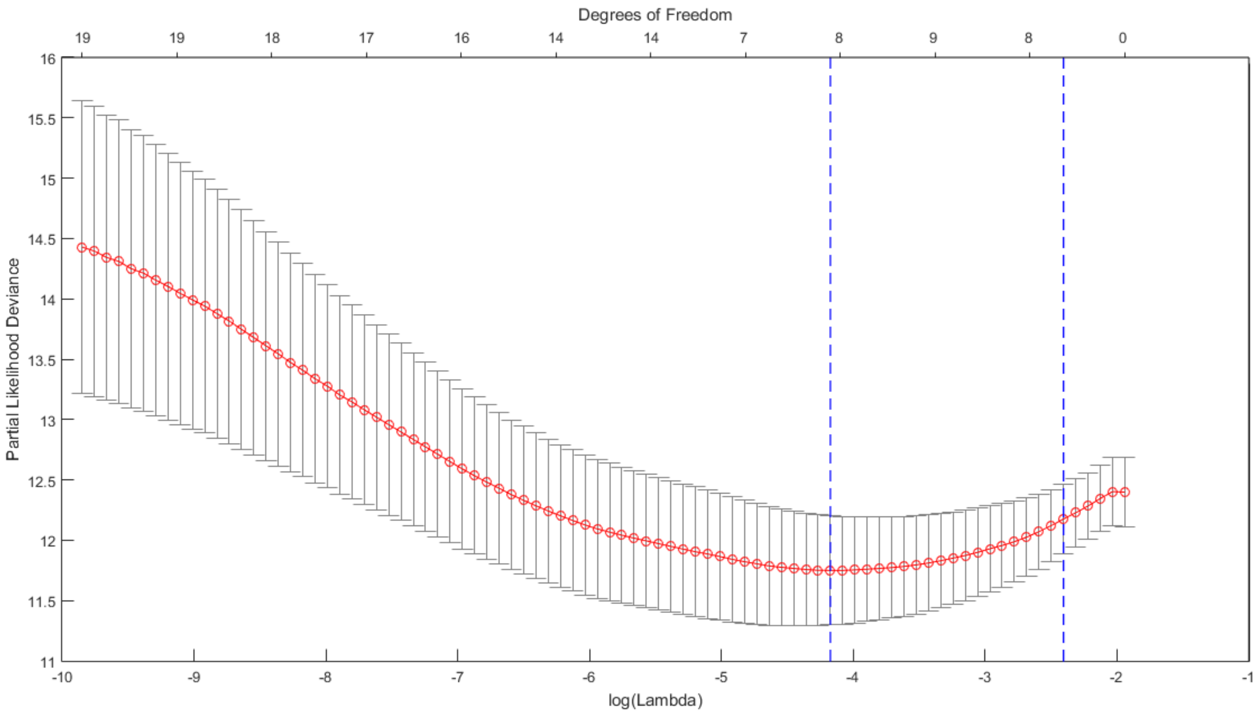

Fig. s2. Cross-validated MSE of the Elastic Net fit
